# Marine Microbiological Enzymes: Studies with Multiple Strategies and Prospects

**DOI:** 10.3390/md14100171

**Published:** 2016-09-22

**Authors:** Yan Wang, Qinghao Song, Xiao-Hua Zhang

**Affiliations:** College of Marine Life Sciences, Ocean University of China, Qingdao 266003, China; wangy12@ouc.edu.cn (Y.W.); 016080910050@sjtu.edu.cn (Q.S.)

**Keywords:** AHL lactonase, polysaccharide-degrading enzymes, marine microorganism

## Abstract

Marine microorganisms produce a series of promising enzymes that have been widely used or are potentially valuable for our daily life. Both classic and newly developed biochemistry technologies have been broadly used to study marine and terrestrial microbiological enzymes. In this brief review, we provide a research update and prospects regarding regulatory mechanisms and related strategies of acyl-homoserine lactones (AHL) lactonase, which is an important but largely unexplored enzyme. We also detail the status and catalytic mechanism of the main types of polysaccharide-degrading enzymes that broadly exist among marine microorganisms but have been poorly explored. In order to facilitate understanding, the regulatory and synthetic biology strategies of terrestrial microorganisms are also mentioned in comparison. We anticipate that this review will provide an outline of multiple strategies for promising marine microbial enzymes and open new avenues for the exploration, engineering and application of various enzymes.

## 1. Introduction

Microorganisms produce series of enzymes [1,2,3]. Given the complicated diversity and ease of large-scale fermentation, microorganisms are widely used in the exploration of enzyme resources. In recent decades, bacteria and fungi from the terrestrial environment have served as the most important and best-studied sources for promising industrial enzymes and secondary metabolites. Recent technological developments have made it easier to utilize marine resources, especially from the deep sea [4,5]. The ocean occupies greater than 70% of total surface of the earth, thus serving as a habitat for numerous microorganisms with vast diversity. The special environmental conditions, involving low temperature, low light, high pressure and high salinity, give marine residents multiple novel characteristic features, which have been attracting increasing attention from marine biologists. Correspondingly, these organisms also produce various novel enzymes and secondary metabolites, some of which have already been used as food additives and potential drugs [6,7,8]. For example, various PKS (polyketide synthase) and NRPS (non-ribosomal peptide synthetase) enzymes responsible for producing secondary metabolites have been identified in marine bacteria, particularly *Streptomyces*, and fungi in recent years [9,10,11,12]. However, compared to terrestrial resources, marine microbial resources, e.g., amylase and alginate lyase, are largely unexplored, although this pool of marine resources is huge. Moreover, the regulatory mechanisms of promising genes and signaling pathway cascades of marine microorganisms are also largely unknown.

In this review, we provide a brief description of two types of promising marine enzymes: acyl-homoserine lactones (AHL) lactonase and polysaccharide-degrading enzymes. We will present the recent research progress regarding these enzymes and discuss potential strategies for further studies. Using well-studied terrestrial microorganisms as references, we hope to open new avenues of exploration, engineering and regulatory mechanisms of marine enzymes.

## 2. AHL Lactonase

### 2.1. Introduction

**Figure 1:**
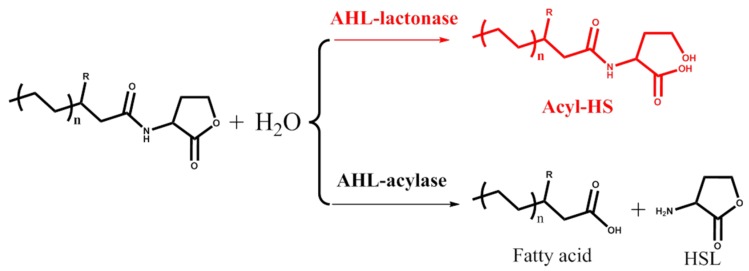
Catalytic mechanism of quorum quenching enzymes.

**Figure 2:**
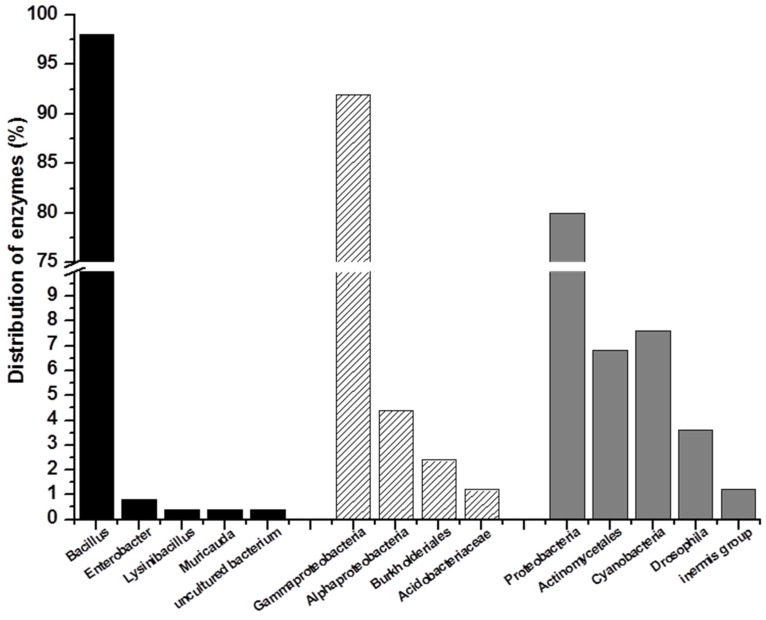
Distribution of AHL lactonase (**left**), amylase (**middle**) and alginate lyase (**right**). Calculation method of the percentage: The reported enzymes were bioinformatic analyzed and classified based on different species source.

Quorum sensing is a population-dependent reaction for microorganisms that occurs via the up/down-regulation of downstream gene expression [13]. The process is also essential for biofilm formation and the secretion of virulence factors, especially in pathogenic bacteria, and causes a series of bacterial diseases [14]. Correspondingly, quorum-quenching technology is an environmentally friendly strategy for disease control [15,16]. AHL lactonase, which degrades molecular *N*-acyl homoserine lactone (AHL) signals, is one of the two types of enzymes involved in quorum quenching (Figure 1). AHL lactonase can open the lactonic ring, and the ring-open molecule is ineffective and cannot reorganize downstream receptor proteins. Several AHL lactonases have been isolated and well-studied in recent years (Table 1). The first reported and well-studied AHL lactonase is AiiA, which inhibits the pathogenic bacterium *Erwinia carotovora* and other plant-related pathogenic bacteria with considerably high activities [17,18]. AiiA is one type of metalloprotein that contains the highly conserved amino acid sequence HXDH-H-D, which serves as a zinc-binding site [17,18]. The conserved sequence is also present in a series of AHL lactonase family members and is essential for normal protein activity [19]. Previous studies revealed that the combination of a yeast strain overexpressing AiiA and the pathogenic bacterium *Aeromonas hydrophila* significantly decreased the death rate of cultivated carp [20,21]. In addition, AiiA, AiiB, AttM, AhlD, QsdA, AiiM and AidH were cloned and characterized from soil *Bacillus*, *Agrobacterium*, *Arthrobacter*, *Rhodococcus*, *Microbacterium* and *Ochrobactrum*, respectively [22,23,24,25,26,27,28]. All of these AHL lactonases were from terrestrial bacteria (Figure 2).

### 2.2. Marine Resources of AHL Lactonase and Research Methods

**Figure 3:**
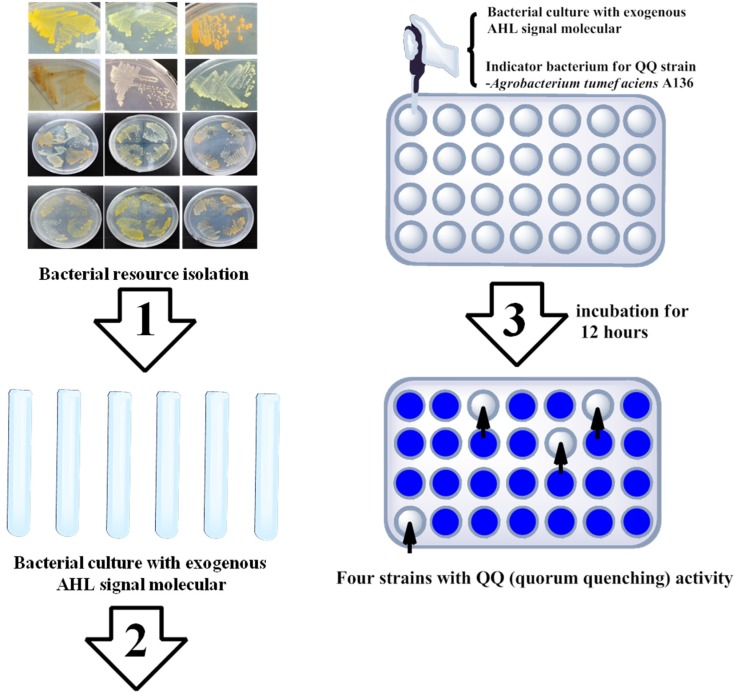
Schematic diagram of high-throughput method for identifying quorum quenching bacteria.

Only a few types of AHL lactonases have been discovered from marine environments. QsdH, which was discovered from marine *Pseudoalteromonas*, is not a single protein but combines with a special transporter of small molecules that belongs to the resistance-nodulation-cell division (RND) superfamily [30]. It is promising to exploit the quorum-quenching enzymes from relatively unknown marine environments. Several new methods have been studied for quorum quenching enzymes exploration. Recently, our group developed a novel high-throughput strategy for identifying bacterial strains with quorum-quenching activity [31]. This method (A136 liquid X-gal (5-bromo-4-chloro-3-indolyl-β-d-galactopyranoside) assay) is based on the measurement of residual AHL molecules after the reaction (Figure 3). Compared with previous strategies, the main improvement of the A136 liquid X-gal assay involves the detection of β-galactosidase activity in a rapid and quantitative manner. Although the ONPG assay (2-nitrophenyl β-d-galactopyranoside) is a broadly approved strategy to measure β-galactosidase activity, the complex process of the ONPG assay causes low throughput and relatively poor efficiency [31]. However, the A136 liquid X-gal assay can be performed in a 96-well plate layout with one-step detection of enzymatic activity in a high-throughput manner. With the A136 liquid X-gal assay, 25 quorum-quenching bacterial strains belonging to different species were identified from hundreds of candidate marine bacterial strains. Additionally, with this method, several genera with quorum quenching activity, such as *Flaviramulus*, *Muricauda* and *Rhodobacter*, were identified [31].

The Gram-negative strain *Muricauda olearia* Th120, which was isolated from *Paralichthys olivaceus*, exhibits high-level quorum-quenching activity. Sequencing and bioinformatic results demonstrate that the genome contains a gene encoding AHL lactonase named MomL. MomL consists of 294 amino acids with a molecular weight of approximately 38.4 KDa [29]. MomL belongs to the metallo-β-lactamase family, and the homolog exhibits a 24.5% similarity with AiiA. In addition to containing the conserved ion-binding site HXDH-H-D, MomL possesses two additional novel characteristic features: (1) the protein possesses one special signal peptide consisting of 21 amino acids at the *N*-terminus of the sequence that presumably facilitate the protein secretory ability; (2) The highest amino acid identity compared with other terrestrial homologs is only approximately 20%. Using the pTWIN1 vector, MomL protein was heterologously-expressed and purified, and the expected approximately 30 KDa band was observed in SDS-PAGE (SDS-polyacrylamide gel electrophoresis). LC-MS (liquid chromatography-mass spectrometry) results demonstrated that MomL degrades C6 or C12-HSL to linear products by hydrolyzing lactonic rings. In vitro enzyme assays indicated that MomL possesses high activity and broad substrate selectivity. Kinetic results indicated MomL had 10-fold increased C6-HSL (C6-homoserine lactones) degrading activity compared with AiiA protein [29].

**Figure 4:**
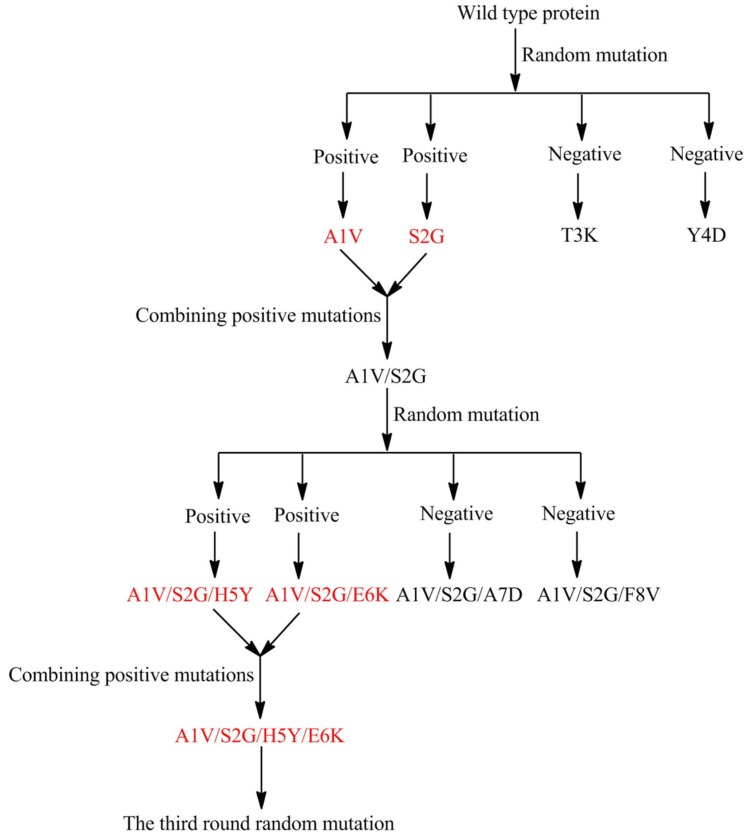
Schematic diagram of directed evolution assay. (A1V means alanine was replaced by valine).

Moreover, using several developed and promising technologies, we performed the following assays using MomL. First, we performed directed evolution of MomL (Figure 4). Although this strategy is not broadly used to engineer marine microbial enzymes, the strategy has been widely used in terrestrial microorganisms. Using directed evolution, Yi Tang’s group at the University of California Los Angeles enhanced the activity of LovD up to 11-fold, which is an acyltransferase that converts the inactive secondary metabolite monacolin J acid into the cholesterol-lowering lovastatin [32]. Regarding the directed mutation strategy, the first step involves constructing a random mutation gene pool of the target protein. To maintain the activity of the mutated protein, the mutation rate was controlled by adjusting the error-prone PCR and sequential error-prone PCR protocols. A mutation rate of 1 to 3 mutated points in every 100 amino acids was suitable for an ideal gene pool [32]. Several screening strategies could be used for positive mutation selection of different proteins with improved activity. These strategies can be easily used for MomL engineering when combined with the high-throughput selection method described above.

Another novel research area for marine microbial enzymes involves gene regulation and signaling cascade pathways. Regulatory studies may aid in the exploration of silencing functional genes and identifying positive regulatory elements of target genes. After identifying positive regulatory genes, another important strategy involves synthetic biology. Through the construction of vectors overexpressing positive regulatory genes, the transcriptional level of the target gene is upregulated, and enzymatic expression is increased. The study of synthetic biology and related research in marine microorganisms is also a relatively new area compared with terrestrial microorganisms. Instead of reviewing marine bacteria, we use soil bacteria as references. *Lysobacter*, a genus of Gram-negative gliding bacteria, has emerged as a novel group of biocontrol agents [33]. Additionally, these species are a new bacterial source of bioactive natural products [34,35]. Liangcheng Du’s laboratory at the University of Nebraska-Lincoln developed a simple method to identify target transformants based on yellow to black color change as a selection marker [36]. Using this special overexpression vector, these researchers constructed various vectors that overexpressed a positive regulator gene identified in the WAP-8294A biosynthetic gene cluster that acted as a potent anti-MRSA (Methicillin-resistant *Staphylococcus aureus*) antibiotic [37], and another regulator gene from the HSAF (Heat Stable Antifungal Factor) biosynthetic gene cluster that acted as an antifungal compound with a novel mode of action [38,39]. The enzymes that produced both WAP-8294A2 and HSAF were upregulated in the strain with overexpressed TonB-dependent receptor, and the production of WAP-8294A and HSAF increased by 2-fold and 7-fold, respectively, compared with the wild type. This work represents a successful metabolic engineering technique in a terrestrial microorganism that can also be used to manipulate unexplored marine bacteria enzyme sources.

**Figure 5:**
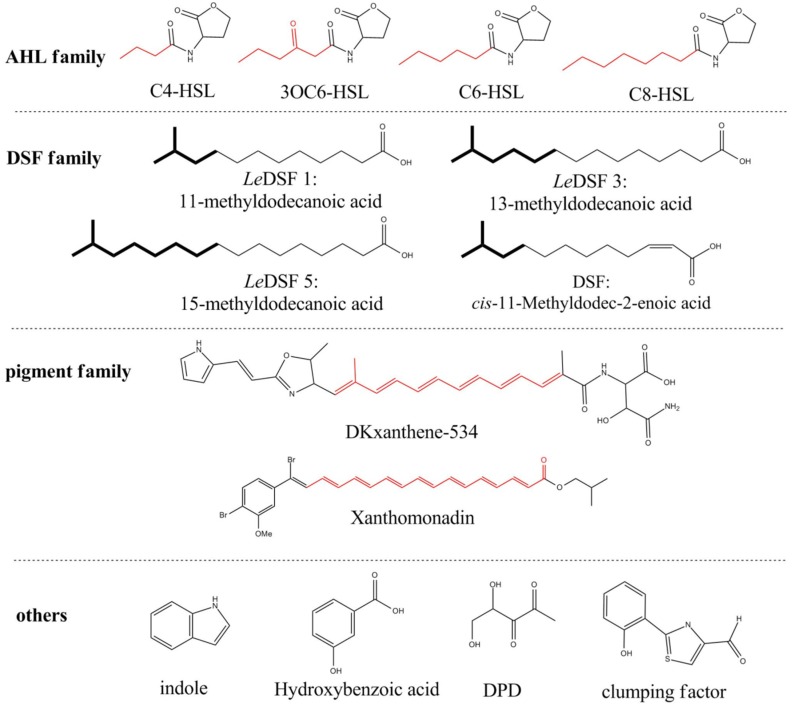
The list of well-known signal molecules.

Signal molecules regulate the transcriptional level of multiple genes, which serves as an additional strategy for exploring functional genes, especially for discovery of silenced gene clusters. This strategy is also relatively newly developed for marine microorganisms compared with soil studies. Recently, with *Lysobacter*, researchers identified a small molecule metabolite (*Le*DSF3) that regulates the biosynthesis of HSAF [40]. The addition of *Le*DSF3 in *L. enzymogenes* cultures increases HSAF biosynthetic gene transcription and HSAF yield. Additionally, the researchers identified the signaling cascade pathway. *Le*DSF3-regulated HSAF transcription and production are dependent on the two-component regulatory system, RpfC/RpfG (histidine kinase sensor/response regulator). Moreover, the global regulator cAMP receptor-like protein, which is a product of the *clp* gene, is another essential element in this signaling cascade pathway [40]. In addition to *Le*DSF3, AHL, indole, diffusible signal factor (DSF), yellow pigments and several other small molecules regulate the transcription of enzyme-encoding genes (Figure 5) [41,42,43,44,45,46]. We have used these strategies to identify the signal cascade of MomL, and the experiment is ongoing with promising progress.

### 2.3. Prospects for AHL Lactonase

Quorum quenching strategy is attracting increased attentions. AHL lactonase, as one kind of quorum quenching enzyme, can be applied in a range of industries. For instance, it can be used in biological control and aquaculture fields to inhibit the toxicities of pathogenic bacterium. It can be used as antistaling agents of fruits and vegetables during long-distance transport. Also, this novel quorum quenching enzyme will be widely applied in antifouling fields to inhibit the formation of bacterial biofilm. Marine-derived AHL lactonase is under highly undeveloped state. Through the updated techniques and methods mentioned above, it is believed that more enzyme resources will be exploited for various applications. 

## 3. Amylase

### 3.1. Introduction

Starch, an important component of the human diet, is one of the main energy storage forms of commercial crops, such as wheat, rice, corn, potatoes and cassava. Based on structure, starches are categorized as amylose and amylopectin. Amylose is linked by a α-1,4 glucosidic bond, whereas amylopectin is linked by a α-1,6 branch bond as well as a α-1,4 linked bond [47]. Currently, acid-catalyzed hydrolysis and enzyme-catalyzed hydrolysis are the main methods of amylo-degradation. Compared with acid-catalyzed hydrolysis, enzyme-catalyzed hydrolysis exhibits considerable advantages, such as substrate specificity and low-energy consumption. Here we listed the properties of well-studied and representative alpha-amylases (Table 2).

### 3.2. Marine Resources of Amylase and Related Catalytic Mechanisms

Currently, heat-stable α-amylases have been well studied and applied in a series of industrial fields. As the development of industry, the demand for cold-active amylase is attracting increasingly attention. Different from terrestrial resources of amylase, many of the marine resources of amylase has high activity in low temperature as over 75% of the ocean is in 0–6 °C. Therefore, the marine environment is an ideal area to find cold-active amylase. In Table 2, we can clearly find that the optimum temperature of α-amylase from *Alteromonas haloplanctis* A23, isolated from Antarctica, is 25 °C and it has high activity in low temperature. In deep sea sediment of Prydz Bay, Antarctic, a cold-adapted α-amylase from *Nocardiopsis* sp. 7326 was identified. It can retain 38% of its highest activity at 0 °C. Recently, our group found a novel alkalophilic α-amylase LaaA which has the highest specific activity reported. The specific activity reached highly to 8881 U/mg. it was cloned from deep-sea bacterium *Luteimonas abyssi* XH031^T^ which isolated from the sediment of the South Pacific Gyre with low temperature. It even maintained 38% residual activity at 10 °C [48].

Given the convenience of genetic and molecular biological manipulation, prokaryotes are the most important resource for α-amylase exploration (Figure 2). Previous studies demonstrated that α-amylase consists of three domains: A, B and C [85]. Domain A, which includes active site residues and is directly related to catalytic reaction, forms the core portion of α-amylase. Domains B and C are related to substrate specificity and active site stability [86]. Thermostability is one of the most valuable characteristic features of amylase. Studies have demonstrated that protein thermostability is mainly influenced by hydrogen bonding, hydrophobic interaction, electrostatic interactions and packing [87]. Through studies of α-amylases from *Bacillus*, two stages of thermo-inactivation have been revealed: the partial unfolded state, which is a reversible step, and the fully unfolded state, which is an irreversible step. With increasing temperatures, the enzyme reaches the partial unfolded state first. When the temperature reaches a certain level, the enzyme is fully unfolded and totally inactive [88]. It is hypothesized that the irreversible inactivation is due to covalent modifications of polypeptide chains or that a higher energy barrier is required during the folding process [86].

To date, most of the well-studied α-amylases contain a conserved calcium site, which is potentially related to enzyme stability and activity [89]. The conserved calcium site is located far away from the active site; thus, this site does not participate in the catalytic reaction but plays an important structural role in enzyme stability and activity [86]. The conserved calcium ion interacts with four amino acid residues, and three of them are strictly conserved in both structure and sequence [90]. Asn104, Asp200 and His235 are three conserved sites in *Bacillus licheniformis* α-amylase (BLA) [91]. Mischa et al. [90] first equivocally elucidated the mechanism of calcium-activating α-amylase, and this group identified a large region that contains 21 disordered residues. The disordered to ordered transition that occurs in this region is mediated by calcium, which leads to the formation of one wall of the cleft containing the extended substrate binding site [90]. If this region is disrupted by two extra residues (Glu-Gly), the conformation of calcium binding is also modified, and thermal stability is subsequently decreased [92].

Great efforts have been made during recent decades through site-directed mutagenesis and directed evolution to improve the properties of promising α-amylases, such as catalytic activity, oxidation resistance, pH tolerance, and temperature tolerance. Directed evolution mainly applies to the enzymes with biochemical and structural properties that remain poorly understood. Site-directed mutagenesis is based on thorough research of enzymatic properties. Using homologous sequence alignment or information regarding tertiary structure, enzymes can be purposefully modified. For example, to improve the thermal stability of α-amylase from *Bacillus megaterium* WHO (BMW), researchers compared BMW-amylase with the most similar protein (*Halothermothrix orenii* α-amylase, 67%) through bioinformatic methods and modified the protein using site-specific mutagenesis. The thermal stability was dramatically improved by H58I mutation, which corresponds to Ile50 in *H. orenii* α-amylase [93].

The deletion of residues is an effective method to improve thermostability. Studies demonstrated that BLA and BAA (*Bacillus amyloliquefaciens* α-amylase) are highly similar in structure, but a significant difference in thermostability was noted. The sequence alignment of BAA and BLA demonstrated the absence of two amino acids, 209E and 210G, in BLA compared with BAA. Then, the mutant strain BAA-△EG (209E and 210G were mutated) was constructed. The results demonstrated that the maximal thermostability was increased by ten degrees compared with the wild type BAA [92]. Similar studies by Mamdouh et al. [94] reported that the amylase of *Bacillus stearothermophilus* US100 has an additional loop compared with the model of BLA. The deletion of two residues (Ile214 and Gly215) increased thermostability and reduced calcium requirements. Further studies revealed that the stability of the loop affects the thermostability. The additional loop (containing residues Gly213, Ile214 and Gly215) along with the neighboring residues Arg212 and Lys216 play a critical role in stabilizing the structure of α-amylase and the calcium-binding site of calcium I. The structure was stabilized via the interaction between Lys216 and Phe194 and Asp238. Moreover, the stability of Lys216 is directly related to the stability of the GIG loop [95]. Hydrogen bonding and salt bridges have the important function of maintaining the stability of α-amylase at low pH [96,97,98]. The stability of mutants at low pH was particularly increased by H275D, H293D and H310D mutations. Histidine (His) is a basic amino acid with positive charges, whereas aspartic acid (Asp) is an acidic amino acid with negative charges. The survey demonstrates that Asp can stabilize the structure of α-amylase by interacting with hydrogen bonding and salt bridges under acidic conditions [99]. Salt bridges are also related by the high thermal stability of BLA [97]. The lysine residues (Lys88, Lys253 and Lys385) interact with each other to form a stable salt bridge [97]. It is proposed that the interaction between two residues with similar charges enhances protein stability. Karimeh et al. [100] found that P407H mutations improved the thermal stability of BAA. Regarding mutated BAA, His407 is located in calcium III, which forms a His-His pair with the neighboring amino acid His406.

### 3.3. Prospects for Amylase

α-amylase is widely applied in food, fermentation and detergent industries. Furthermore, in the medical field, α-amylase can be used as drug targets for treating diabetes, obesity and high cholesterol, etc. As the increasing demand of different special properties of α-amylase in the industry and research fields, the complex marine environments provide the possibility of finding various of α-amylases. The study of marine resources of amylase is becoming increasingly popular. Besides, protein engineering is an efficient method to improve the properties of α-amylase and plays an increasingly important role in the research of α-amylase.

## 4. Alginate Lyase

### 4.1. Introduction

**Figure 6:**
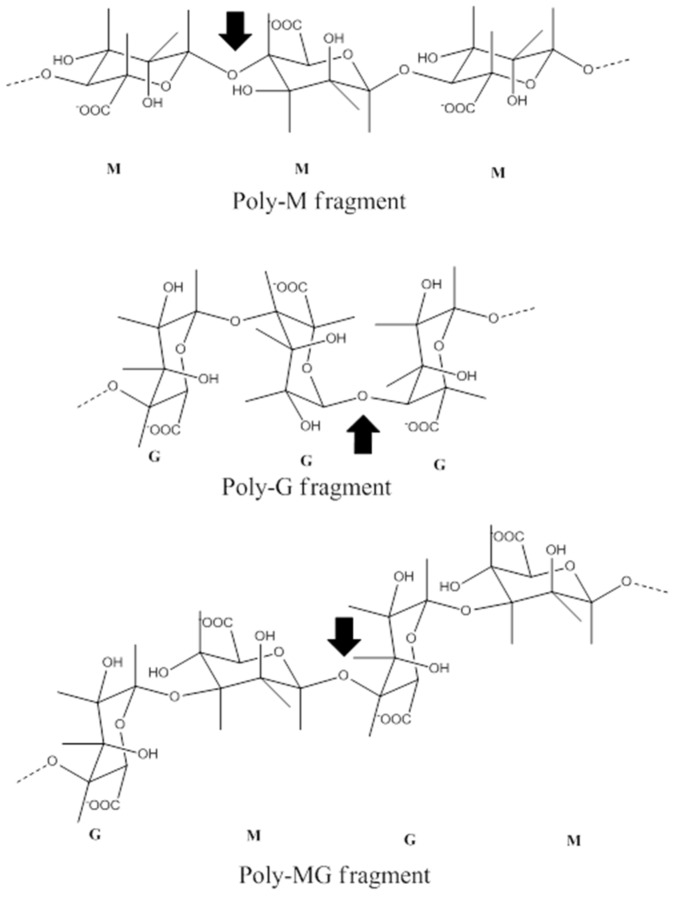
Structure of algin and the mode of action of alginate lyase.

Algin is a linear complex copolymer composed of mannuronate acid (M) and guluronic acid (G) that was originally extracted from the mesenchyme of kelp, gulfweed, and seaweed (Figure 6). Alginate lyase degradation products include oligosaccharides, which exhibit various bioactivities, such as antibiosis, anti-cancer, anti-tumor and promoting plant growth. Theses characteristic features allow these proteins to have broad application prospects in medical and agricultural fields. The methods of algin degradation include acid hydrolysis, chemical oxidation and enzymolysis, among which enzymolysis has great potential usage given its high specificity and reaction efficiency.

Alginate lyase is a type of enzyme that specifically degrades algin and is primarily found in microorganisms, animals and plants. Most bacterial alginate lyases are from *Gamma-proteobacteria* (Figure 2). Alginate lyases derived from microorganisms are more stable and exhibit increased activity compared with those obtained from plants and animals. Various types of classification have been reported, including those based on the specificity of the degradation substrate. In addition, algin enzymes can be classified as a mannuronate acid lyase, which exclusively degrades the M section, or a guluronic acid lyase, which exclusively degrades the G section (Figure 6). At present, most of the well-studied alginate lyases exhibit M section-degrading activity, whereas a small proportion degrades both M and G sections (Figure 6) [101].

### 4.2. Marine Resources of Alginate Lyase and Catalytic Mechanisms

Given the special environment and promising potential, marine alginate lyase studies have become common in the last several decades. Tseng and colleagues isolated two types of alginate lyases from *Vibrio* sp. AL-9 in 1992. One type breaks the α-1,4 glycosidic bond of guluronic acid, whereas the other type breaks the β-1,4 glycosidic bond of mannuronate acid [102]. Moreover, this group also identified an alginate lyase that degrades guluronic acid in *Vibrio* sp. AL-128 [103]. The alginate lyase isolated from *Vibrio* sp. QY101 by Song et al. [104] exhibited obvious activity for both mannuronate acid and guluronic acid. For the first time, this group reported that the 9-amino acid region (YXRESLREM) appears not only in guluronic acid lyase but also in mannuronate acid lyase [105]. Takeshita et al. identified one novel alginate lyase from a *Vibrio* species isolated from the intestinal tract of red snapper [106]. The special alginate lyase retains 45% of catalytic activity under heat shock conditions of 100 °C. Moreover, activity is also retained after treatment with 3% SDS in 25 °C for 30 min [106].

In 2009, Liu et al. [107] constructed the recombinant plasmid pINA1317-Y1CWP110 with alginate lyase (AlyVI) isolated from *Vibrio* sp. QY101 as the target gene. This enzyme has considerable catalytic degradation activity if expressed in *Yarrowia lipolytica.* Moreover, this enzyme degrades mannuronate acid, guluronic acid, and alginate and produces a series of oligos with different lengths [107]. Alginate lyase was expressed in *Saccharomycetes* for the first time, and different oligosaccharide lengths were produced. In the bacterial strain *Pseudoalteromonas atlantica* AR06, some research groups used homologous recombination technology to fuse green fluorescent protein (GFP) to the *C*-terminus of alginate lyase AlyA, demonstrating that the bacterial strain has normal degradation activity and is able to release fluorescence. As a visible gene expression tool, GFP is convenient for biochemical and catalytic studies of alginate lyase. Recently, several additional alginate lyases have been well studied. Liu et al. [108] reported for the first time that the extracellular alginate lyase-like protein from *Pseudomonas fluorescens* exhibits high degradation activity for alginate. Dong and other scientists performed a study on bacteria isolated from the Arctic marine seaweed that produces alginate lyase [109]. In total, 65 bacterial strains were isolated from the kelp specimen, 21 of which exhibited alginate lyase activity. Among these isolates, 11 bacterial strains exhibited an optimum temperature between 20 °C and 30 °C, which indicates that the extracellular lyases are cryophilic enzymes [109]. Moreover, the bacterial strains *Psychrobacter*, *Winogradskyella*, *Psychromonas* and *Polaribacter* produce alginate lyase. We have recently focused our studies on *Luteimonas abyssi* sp. nov., which was isolated from the abyssal sediment under the circulating area of the South Pacific Ocean. Given that the strain lives in low-temperature environments and possesses alginate lyase activity, we hypothesize that alginate lyase is a cold-adaptive enzyme.

Previous studies identified different degradation products of alginate lyase under different pH conditions. For example, vAL-1 from PL-14 exhibits glucuronic acid degradation activity. In addition, this enzyme also degrades algin under alkaline conditions [110]. The purified alginate lyase Smlt1473 from *S. maltophilia* inducing exhibits hyaluronic acid degradation activity at a pH of 5, guluronic acid degradation activity at a pH of 7 and alginate degradation activity at a pH of 9 [111].

Secondly, according to the similarity of amino acid sequence, alginate lyases may be classified into the PL-5, PL-7 and PL-15 families. In the PL-5 family [112], 41 alginate lyases have been identified to date, all of which are from microorganisms. In addition, 86 alginate lyases have been discovered in the PL-7 family, including 84 from bacteria and only 2 from eukaryotic cells. The PL-15 family currently has 11 alginate lyases, and all of them are from bacteria. Thus, most of the identified alginate lyases have been identified in bacteria.

Thirdly, alginate lyases can be divided into three categories based on molecular weight [113]: 20 to 35 kD, approximately 40 kD and approximately 60 kD. The first type of enzyme (20 to 35 kD) exhibits a variety of substrate specificity. Given that the alginate lyases with a molecular weight of approximately 40 kD have degradation activity specific to the M section [114] and most of them contain the common conserved sequence NNHSYW, it is inferred that this sequence is related to the M section-degradation activity. The homologous sequence YFKAGXYXQ of the *C*-terminus is another characteristic feature of small alginate lyases [101]. However, this same sequence was recently identified in AlyPI lyase, which is a member of the 60-kD family [115]. By comparing the homology of abalone alginate lyases (HdAly) with members of the PL-14 family, such as turban shell SP2 and chlorella virus CL2, Sayo Yamamoto et al. [116] reported that Arg92, Lys95, Arg110, Arg119, and Lys19 are particularly highly conservative amino acid residues. Utilizing site-specific mutagenesis technology, it was discovered that Lys95 mutations cause complete enzymatic inactivation, whereas Arg92, Arg110 and Arg119 mutations lead to reductions in activity greater than 65%, thus suggesting that the Arg92 to Arg119 region is closely related to HdAly activity [116].

To date, all of the alginate degradation enzymes have been classified as lyases, and no hydrolytic enzymes have been reported [117]. Alginate lyases catalyze the degradation of alginate via a β-elimination mechanism by breaking α-1,4 glycosidic bonds and yielding oligosaccharides with an unsaturated double bond at the non-reducing end. A portion of the reaction in which alginate lyase is cleaved can be induced by a specific substrate. For example, Matsushima et al. [118,119] reported that alyA gene transcription in *Pseudoalteromonasatlantica* AR06 is induced by alginate in basic medium.

### 4.3. Prospects for Alginate Lyase

Alginate lyases can be utilized in multiple industrial and medical areas. Alginate lyase can be used for developing new methods of tissue regeneration. For example, stem cell cultivation needs alginate hydrogel as a biological support. However, mammals cannot produce alginate lyase, and thus, alginate hydrogel cannot be easily degraded. Theoretically, alginate lyase provides a viable way to solve this problem [120]. Alginate lyase can also improve the drug susceptibility of biofilms, working as quorum sensing inhibitors [121]. Moreover, it has an inhibitory effect on the pathogens of pulmonary cystic fibrosis. *Pediococcus* sp. Ab1, which has alginate lyase activity, can be used as a probiotic to improve the intestinal microflora and nutritional status of abalone during their cultivation [122].

## 5. Chitinase

### 5.1. Introduction

Chitin is an *N*-acetyl-glucosamine homopolymer linked by a β-1,4-glucosidic bond [123] that is structurally identical to cellulose, except that the hydroxyl group in cellulose at C2 is replaced by an acetamide group [124]. Chitin is a particularly rich and important nutrient and energy source for maintaining the ecosystems of marine environments. The majority of chitin originates from marine ecosystems, and chitinolytic marine bacteria play a critical role in recycling chitinous materials, such as exoskeletons of crustaceans and insects. Moreover, chitinolytic marine bacteria are also important to maintain the balance of marine ecosystem. The biofunction of chitin mainly involves its degradation product oligochitosan, which is capable of resisting fungus, tumors, plant diseases, and related pests. Oligochitosan also alters the body’s immunological function. Compared with traditional chemical approach, oligochitosan production by biological methods is milder and more environmentally friendly. Thus, chintinase studies have drawn increased attention during recent decades.

### 5.2. Marine Resources of Chitinase and Catalytic Mechanisms

Currently, chitinases have been identified in a series of marine organisms, such as *Alteromonas* sp. O7, *V. parahaemolyticus* [125], *Salinivibrio costicola* [126] and *Microbulbifer degradans* [127]. Chitinase is responsible for hydrolyzing the β-1,4-glycosidic bond of different types of chitin. Based on catalytic domain similarities, chitinase can be divided into two families: 18 and 19. Family 18 includes chitinases from bacteria, fungi, viruses, animals and some plants, whereas family 19 includes chitinases from most plants and special bacteria, such as chitinase C (ChiC) identified from *Streptomyces griseus* [128]. The chitinases of the two families, which potentially evolved from different ancestors, possess different catalytic mechanisms [129]. The catalytic domain of the GH18 family has a typical triosephosphate isomerase (TIM) structure that forms an inner barrel with 8 α-helixes surrounded by an outer barrel of 8 β-sheets. Whereas the catalytic domain of the GH19 family has a high proportion of α-helixes, and its structure is similar to chitosanase and lysozyme. GH18 contains 3 groups (ChiA/ChiB/ChiC), which are classified according to amino acid sequence differences in the catalytic domain. ChiA and ChiB hydrolyze chitin chains towards opposite direction, whereas ChiC is an endochitinase [130]. Most chitinases from marine microorganisms belong to family 18 [131,132]. Comparing with chitinases isolated from terrestrial bacteria, marine chitinase exhibit better pH and salinity tolerance, which may represent promise for some special applications. Wang et al. [133] isolated marine *Bacillus cereus* that express two chitinases with optimum pH, optimum temperature, pH stability, and thermal stability values of 9, 50 °C, 3 to 11, 50 °C and 5, 40 °C, 3 to 11, 60 °C, respectively. These enzymes retain 61%, 60%, 73%, and 100% as well as 60%, 60%, 71%, and 96% of their original activity in the presence of 2% Tween 20, 2% Tween 40, 2% Triton X-100, and 1 mM SDS, respectively [133]. The marine psychrophilic bacterium isolated by Stefanidi et al. from a sample raised from a depth of 1200 meters in the northern Pacific Ocean, secretes several chitinases in response to chitin induction. These chitinase genes encode a protein of 550 amino acids. The optimum pH and temperature of this chitinase are 5.0 and 28 °C, respectively. There are two crucial residues, Trp275 and Trp397, in the catalytic domain of the chitinase [134]. A chitinase directed mutagenesis study by Suginta et al. [134] revealed that Gly and Phe instead of Trp275 and Trp397, respectively, heavily altered the selection of β substrate isomers. The Trp275 mutation alters the chitinase’s kinetics characteristic features by increasing the catalytic constants (*k*_cat_) and the specificity (*k*_cat_ /*K*_M_) of all substrates 5- to 10-fold. In contrast, the Trp397 mutation decreases the strength of binding between chitinase and substrate and the rate of soluble substrate degradation.

### 5.3. Prospects for Chitinase

The degradation of chitin into oligosaccharides has promise that may be useful in numerous biological functions, such as antimicrobial activity and antitumor activity [135]. At present, chitooligosaccharides are mainly produced through chemical reactions in industry. This process has many drawbacks, such as the production of a series of unexpected short strain oligosaccharides. Moreover, chemical reactions can easily cause serious environmental pollution. Comparatively, biological degradation using chitinase has many advantages, such as being environmentally friendly, inexpensive and repeatable. Because of these advantages, the use of chitinase to hydrolyze chitin has drawn increasing attention during recent years. 

## 6. Cellulase

### 6.1. Introduction

Cellulases are multi-component enzymes that can be divided into three components based on catalytic function, including endoglucanase (endo-1,4-β-d-glucanase, EC3.2.1.4), exoglucanase (exo-1,4-β-d-glucanase, EC3.2.1.91), and cellobiase (β-1,4-glucosidase, EC3.2.1.21). Cellulases exhibit great potential in various applications, including papermaking, detergents, bioenergy and effluent treatment. Previous studies were limited to terrestrial-derived cellulases. As research on marine microbes and their enzymes advance, it was discovered that enzymes secreted by marine microbes possess several characteristic features, such as pressure tolerance, alkali resistance, cold resistance and heat resistance. As a result, marine cellulase resources have drawn increasing attention [136,137].

### 6.2. Marine Resources of Cellulase and Catalytic Mechanisms

Thermophiles and cryophiles are two main types of marine microbes in the research of cellulose. For example, *Rhodothermus marinus* and *Thermotoga neapolitana* are well characterized. Extreme thermophile cellulose is composed of several domains. The cellulose and hemi-cellulase domains are catalytic domains that are linked by several cellulose binding domains (CBDs) [138]. *Rhodothermus marinus* express a type of cellulose with an optimal temperature that is greater than 90 °C. Bioinformatics and three-dimensional structure comparisons suggest that the aromatic amino acid cluster exposed on the surface of the protein is responsible for its thermophile activity [139]. Hakamada and coworkers [140] analyzed the structures of thermophile cellulose and alkali cellulose and revealed that three lysines located at 137, 179 and 194 are responsible for heat resistance. Moreover, they also demonstrated that increases in arginine, histidine and glutamine residues and decreases in aspartate and lysine residues are also related with alkali cellulose stability. *Pseudoalteromonas* sp. is another species gaining considerable attention. The optimal temperature of its cellulose, which ranges from 45 to 60 °C, is lower than that of other microbial celluloses. Lee and his partner [141] isolated a type of marine bacteria *Bacillus subtilis*. The optimal temperature of its cellulose is 50 °C, and the optimum reaction pH is 6.5. Alfredsson reported on the marine bacteria *Rhodothermus marinus* isolated from alkaline underwater hot springs in Iceland. The optimum growth conditions of the enzyme are 65 °C, pH 7.0 and 2% NaCl [142]. Trivedi [143] reported on *Bacillus flexus* from alga that produces alkaline cellulose. The molecular mass of the cellulose is 97 kDa, and good stability is noted at pH ranges of 9.0 to 12.0. Moreover, approximately 70% of activity is maintained in 15% NaCl. Fellerand and his co-workers [51] isolated the psychrophilic filamentous bacterium near the Zhongshan Station and the Great Wall Station. This species produces cellulase and decomposes cellulase at 0 or 5 °C, thus maintaining proliferation at low temperatures [51]. Recently, Fang [144] identified a β-glucosidase gene named *bgl1A* from a marine microbial metagenomic library by functional screening. This gene permitted tolerance of high glucose concentrations. The protein BgllA was identified as a member of the glycoside hydrolase 1 family. The recombinant β-glucosidase Bgl1A exhibited a high level of stability in the presence of various cations and high concentrations of NaCl. The protein was activated by glucose with low concentrations. The enzymatic activity of Bgl1A was gradually inhibited by increasing concentrations of glucose, but 50% of the original value remained even in up to 1000 mM glucose.

### 6.3. Prospects for Cellulase

Cellulases from marine microorganisms exhibit activities under extreme conditions, such as high salt, high pressure, pH and high/low temperature. Thus, microorganisms abundant in the unique marine environment provide an important material base for exploiting new source of cellulases. For example, alkaline cellulose is used in the detergent industry. Moreover, alkaline celluloses exhibit more advantages in the disposal of sewage from spinning, papermaking, pickling and sauce production. Moreover, laundry processes require cellulases with the properties of alkali resistance, heat resistance and insensitivity to surfactants to simultaneously cut losses in detergent processing, storing and transiting. With the rapid development of the seaweed industry, there is an enormous demand for cellulases used in algae wall solutions and degradation of algae processing wastes. With the development of biotechnologies, especially bioinformatics and metagenomics, we believe that more cellulases with important functions that were previously unable to be discovered will be exploited in the future.

## 7. Conclusions

Marine microorganisms contain a series of novel and studied enzymes. However, due to limitations of exploration, a large proportion of these organisms have not been identified. This review presents several classic methods for enzyme transcriptional regulation and engineering, which can be used in marine microbial enzyme exploration. Additionally, this review describes the mechanism and current status of several polysaccharide-degrading enzymes. Hopefully, these novel strategies and well-studied catalytic mechanisms can serve as a reference for identifying novel enzymes from marine environments.

## Figures and Tables

**Table 1:** Properties of well-studied acyl-homoserine lactones (AHL) lactonase.

QQ Enzyme	Length (aa)	Predictable Domains	Signal Peptide	Host Organisms	Origin	Structure	Reference
AiiA	231aa	Beta-lactamase family (15–216)	No signal	*Bacillus*	terrestrial	3DHB	[18]
AiiB	276aa	Beta-lactamase family (42–259)	No signal	*Agrobacterium*	terrestrial	unknown	[22]
AttM	295aa	Beta-lactamase family (78–282)	1–17	*Agrobacterium*	terrestrial	unknown	[25]
QsdA	323aa	Phosphotriesterase family (11–322)	No signal	*Rhodococcus*	terrestrial	unknown	[24]
AidH	279aa	Alpha/beta hydrolase (25–147)	No signal	*Ochrobactrum*	terrestrial	unknown	[27,28]
GKL	330aa	Phosphotriesterase family (16–329)	No signal	*Geobacillus*	terrestrial	unknown	[26]
MomL	293aa	Beta-lactamase family (72–277)	1–21	*Muricauda*	oceanic	unknown	[29]
QsdH	968aa	AcrB/AcrD/AcrF family (182–964)	1–23	*Pseudoalteromonas*	oceanic	unknown	[30]

**Table 2:** Properties of well-studied alpha-amylases.

Stain	UniProtKB	Molecular Mass (kDa)	Signal Peptide (aa)	Temperature Optimum (°C)	Thermostabiliy	pH Optimum	pH Stability	Specific Activity with Soluble Starch (U/mg)	References
*Luteimonas abyssi*	NM	49	35	50	34%, 50 °C, 20 min	9	>50%, 6–11, 50 °C, 1 h	8881 ^a^	[48]
*Bacillus licheniformis*	Q208A7	55	29	90	Clear halos, 100 °C, 120 min	NM	NM	NM	[49]
*Bacillus amyloliquefaciens*	P00692	54.8	31	60	NM	NM	NM	NM	[50]
*Alteromonas haloplanctis* A23	P29957	50	24	25	6%, 25 °C	7	NM	NM	[51]
*Bacillus* sp. strain KSM-K38	Q93I48	55	21	55–60	20%, 50 °C, 30 min	8.0–9.5	>80%, 6–11, 40 °C, 30 min	4221 ^a^	[52]
*alkaliphilic* bacterium N10	Q6WUB6	61	31	50	71%, 50 °C, 30 min	9.5	>80%, 8.5–11, 50 °C, 10 min	7826 ^a^	[53]
*Bacillus* sp. XAL601	Q45643	225	31	70	NM	9.0	NM	57.3 ^a^	[54]
*Bacillus* sp.	O82839	53	31	55	25%, 80 °C, 10 min	8.0–8.5	>50%, 6–9, 40 °C, 30 min	5009 ^a^	[55]
*Nocardiopsis* sp. 7326	NM	55	NM	35	18%, 55 °C, 30 min	8.0	>60%, 7–9, 4 °C, 24 h	548 ^a^	[56]
*Bacillus* sp. strain GM8901	NM	97	NM	60	37%, 60 °C, 2 h (−Ca), 78%, 60 °C, 2 h (+Ca)	11–12	>85%, 6–13, 50 °C, 1 h	157.5 ^a^	[57]
*Bacillus* sp. NRRL B-3881	NM	NM	NM	50	50%, 55 °C	9.2	>50%, 7.0–10.5	3485 ^a^	[58]
*Bacillus acidicola*	J9PQD2	62	no signal	60	50%, 90 °C, 10 min	4	100%, 4, 12 h, 100%, 3, 1 h	1166 ^a^	[59]
*Lipomyces kononenkoae*	Q01117	76	28	70	0, 70 °C, 10 min	4.5–5.0	>70%, 3–8, 1 h	258 ^a^	[60]
*Alicyclobacillus acidocaldarius*	C8WUR2	160	23	75	NM	3	NM	16.9 ^b^	[61]
*Bacillus* sp. *Ferdowsicous*	P86331	53	NM	70	75%, 75 °C, 45 min	4.5	>75%, 3.5–6, 60 min	267 ^a^	[62]
*Bacillus acidocaldarius*	NM	68	NM	75	50%, 60 °C, 5 days	3.5	Stable below 4.5	257 ^b^	[63]
*Aspergillus penicillioides*	NM	42	NM	80	60%, 100 °C	9	>80%, 7–10	118.42 ^a^	[64]
*Talaromyces pinophilus* 1–95	NM	58	NM	55	<45 °C, 1 h	4–5	5–9.5, 24 h	673.08 ^a^	[65]
*Thermococcus* sp. HJ21	B4X9V8	51.4	NM	95	50%, 90 °C, 5 h, 40%, 30%; 100 °C; 2 h, 3 h	5	5–9	8.3 ^a^	[66]
*Malbranchea cinnamomea*	K9L8F3	60.3	21	65	50%, 60 °C, 41.1 min	6.5	>90%, 5–10, 30 min	514.6 ^a^	[67]
*Aspergillus niveus*	NM	60	NM	65	50%, 70 °C, 20 m	6	4–7, 24 h	168 ^a^	[68]
*Thermoactinomyces vulgaris*	G8ZE61	40.6	NM	50	50%, 50 °C, 2 h	6–7	4–9	127,100.33 ^b^	[69]
*Bacillus* sp. AAH-31	S6BGD1	91	28	70	<60 °C	8.5	6.4–10.3	16.7 ^a^	[70]
*Paecilomyces variotii*	NM	75	NM	60	50%, 60 °C, 53 min	4	>70%, 5–8, 1 h	612.5 ^a^	[71]
*Pseudoalteromonas arctica* GS230	NM	55	24	30	49%, 30 °C, 150 min	7.5	>60%, 7–8.5, 1 h	25.5 ^a^	[72]
*Bacillus* sp. YX-1	A9YDD9	56	31	40–50	60%, 60 °C, 1 h	5	>80%, 4.5–11, 1 h	607 ^b^	[73]
*Geobacillus thermoleovorans*	NM	26	NM	100	50%, 100 °C, 3.6 h	8	50%, 6, 4.5 h, 50%, 7, 7.5 h	450 ^a^	[74]
*Fusicoccum* sp. BCC4124	Q0Z8K1	50	no signal	70	95%, 50 °C, 1 h	7	NM	90 ^a^	[75]
*Bacillus subtilis* KCC103	A8VWC5	53	33	65–70	50%, 70 °C, 7 min	6–7	>98%, 5–9.5	483 ^a^	[76]
*Bacillus subtilis* AX20	NM	149	NM	55	50 °C, 30 min	6	5–9, 24 h	4133 ^a^	[77]
*Halothermothrix orenii*	Q8GPL8	60	23	65	37–75 °C	7.5	6–9.5	22.32 ^a^	[78]
*Bacillus stearothermophilus*	NM	64	NM	50	92%, 100 °C, 1 h	7	23%, 3, 1 h, 26%, 10, 1 h	77.2 ^b^	[79]
*Thermus filiformis* Ork A2	NM	60	NM	95	50%, 95 °C, 19 min	5.5–6	>80%, 4.5–8, 1 h	6352 ^b^	[80]
*Bacillus subtilis*	NM	48	NM	50	70%, 60 °C, 1 h	6.5	5–6.5	772.7 ^a^	[81]
*Clostridium perfringens* NCTC 8679	NM	76	NM	30	NM	6.5	NM	NM	[82]
*Escherichia coli* (strain K12)	P25718	75.7	17	NM	NM	8	NM	NM	[83]
*Bacillus subtilis*	P00691	67	27	NM	NM	8.5	NM	NM	[84]

^a^ Enzyme activity are measured by DNS method; ^b^ Enzyme activity are measured by the colored starch-I_2_ complex method; NM means not mention in the essays.
